# In-hospital initiation of PCSK9 inhibitor and short-term lipid control in patients with acute myocardial infarction

**DOI:** 10.1186/s12944-022-01724-9

**Published:** 2022-10-24

**Authors:** Bowen Lou, Hui Liu, Yongbai Luo, Gulinigaer Tuerhong Jiang, Haoyu Wu, Chen Wang, Yue Wu, Bo Zhou, Zuyi Yuan, Jianqing She, Junhui Liu

**Affiliations:** 1 Cardiovascular Department, the First Affiliated Hospital of Xi’an Jiaotong University, 277 West Yanta Road, Xi’an, 710061 Shaanxi China; 2 Key Laboratory of Environment and Genes Related to Diseases, Ministry of Education, Xi’an, 710061 Shaanxi China; 3 Biobank, First Affiliated Hospital of Xi’an Jiaotong University, Xi’an, 710061 Shaanxi China; 4 Respiratory Department, First Affiliated Hospital of Xi’an Jiaotong University, Xi’an, 710061 Shaanxi China; 5 Diagnostic Department, First Affiliated Hospital of Xi’an Jiaotong University, Xi’an, 710061 Shaanxi China

**Keywords:** Proprotein convertase Subtilisin/kexin type 9 (PCSK9) inhibitors, Statin, Low density lipoprotein, Acute myocardial infarction

## Abstract

**Background:**

Proprotein convertase subtilisin/kexin type 9 (PCSK9) inhibitors have been shown to improve cardiovascular outcomes when added to conventional statin therapy. This study aims to investigate the efficacy and safety of in-hospital initiation of PCSK9 inhibitors among patients with acute myocardial infarction (AMI) based on real-world experience.

**Methods and results:**

Data were collected from the Biobank of the First Affiliated Hospital of Xi’an Jiaotong University between January 2016 and December 2020. A total of 7556 AMI patients were screened for eligibility. Propensity Score Match (PSM) was employed, and covariates were age, sex, admission blood pressure and lipid profiles.

Eligible participants were (1) propensity-matched 1:2:2 of statin plus evolocumab (dual therapy) vs. statin vs. statin plus ezetimibe. Ninety-five statin plus evolocumab users achieved significantly decreased low density lipoprotein (LDL) levels (0.92 ± 0.62 mmol/L in the 1st month and 1.17 ± 0.73 in the 3rd month) and a promising attainment rate of LDL (79.5% in the 1st month and 80.0% in the 3rd month) compared to the other two groups. (2) Propensity-matched 1:2:2 of statin plus ezetimibe evolocumab (triple therapy) vs. statin vs. statin plus ezetimibe. Similarly, 75 triple medication users achieved significantly decreased LDL levels and a promising attainment rate of LDL compared to the other two groups. In-hospital mortality and readmission rates within 3 months were then analyzed, and a decreased readmission rate was observed with PCSK9i therapy.

**Conclusions:**

Based on the present single-center real-world PSM-adjusted study, PCSK9i has been effective in short-term lipid control among AMI patients. The long-term effectiveness for reducing major cardiovascular events among AMI patients based on real-world experience remains to be explored.

**Trial registration:**

The study was registered at ClinicalTrials.gov, ClinicalTrials.gov ID: NCT05184530

**Graphical Abstract:**

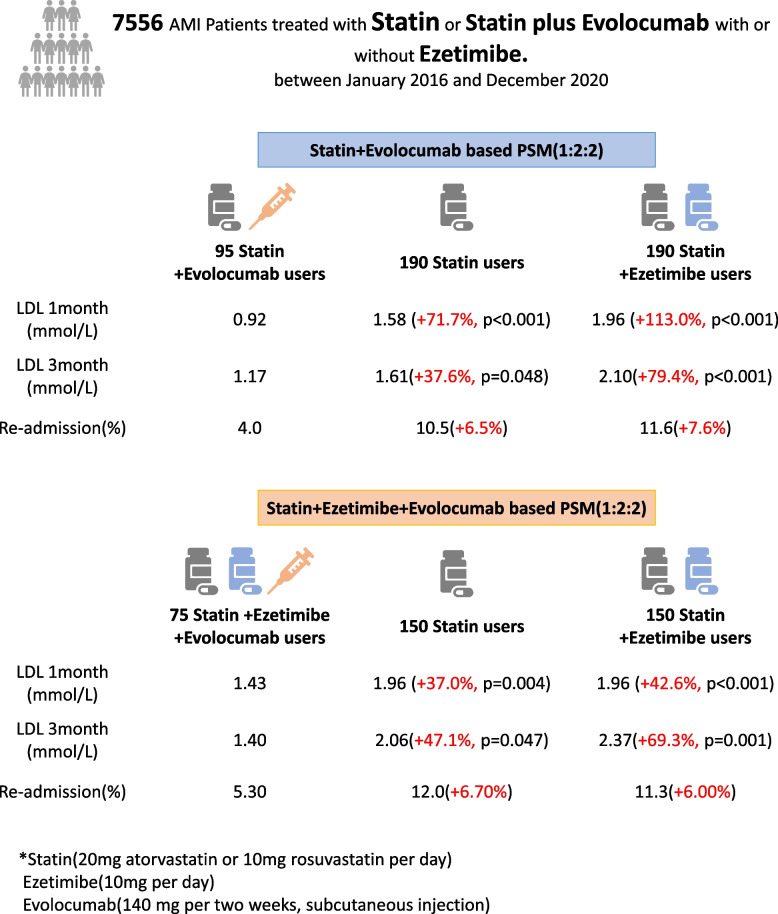

**Supplementary Information:**

The online version contains supplementary material available at 10.1186/s12944-022-01724-9.

## Introduction

The advent of the Proprotein Convertase Subtilisin-Kexin Type 9 (PCSK9) has brought a breakthrough in the treatment of atherosclerotic disease. PCSK9 is a secreted serine protease synthesized by the liver that can bind to and degrade LDL receptors, thereby reducing the clearance of serum LDL [1]. Previous clinical studies have shown that patients with atherosclerotic disease still have poor LDL-C level control under conventional statin and ezetimibe treatment. The recommended LDL target values for ASCVD patients tend to be lower and lower; currently, for patients at very high risk, an LDL reduction of ≥50% from baseline and an LDL goal of < 1.4 mmol/L (< 55 mg/dL) are suggested [2, 3]. A growing number of clinical studies of PCSK9i have proven that high-risk atherosclerosis patients can achieve satisfactory blood lipid (especially LDL-C) control by combining PCSK9i on the basis of statin treatment, thus further reducing the risk of myocardial infarction, stroke and other events [4–6].

According to contemporary guidelines [7, 8], PCSKi should be initiated when the initiation of statin and ezetimibe has not met the targeted LDL-C levels. However, few studies have focused on the effectiveness, safety and cardiovascular benefits of PCSK9i on LDL-C management when initiated during the acute phase of cardiovascular events. In the latest Chinese consensus on dyslipidemia management in patients with high-risk atherosclerosis disease [9], it is recommended that individualized lipid-lowering therapy should be selected according to the patient’s baseline LDL-C level and the expected reduction range of the lipid-lowering regime. As a result, some AMI patients with high baseline LDL-C levels might opt to receive the combined treatment of statins and PCSK9i in the early stage of AMI onset. However, clinical evidence for the efficacy and safety of this treatment regimen that combined PCSKi and statins could be initiated during the acute phase of AMI for high-risk patients with high baseline LDL-C levels based on real-world experience is still limited.

Therefore, this study aims to investigate the efficacy and safety of in-hospital initiation of PCSK9i in patients with AMI based on real-world experience. Through this single-center real-world study, in-hospital initiation of PCSK9i was shown to be effective in short-term lipid control among AMI patients. The long-term effectiveness for reducing major cardiovascular events remains to be explored.

## Methods

### Study design and clinical data collection

This was a single-center, retrospective cohort study. A total of 7556 consecutive patients admitted to the cardiology department of the First Affiliated Hospital of Xi’an Jiaotong University for AMI between January 2016 and December 2020 were enrolled. The inclusion criteria were confirmed admission diagnosis of AMI and were defined based on the universal definition criteria by the American Cardiology College [10]. The exclusion criteria were as follows: (1) severe noncardiac disease with an expected survival of less than 1 year and unwillingness to participate; (2) patients over the age of 80 years or living far away from the hospital’s catchment area. A patient could only be included once. In hospitals, PCSK9i was initiated according to the Chinese consensus on dyslipidemia management in patients with high-risk atherosclerosis disease [9].

The medical records of the patients were collected from the Biobank of the First Affiliated Hospital of Xi’an Jiaotong University, which contains the identified data derived from raw medical records, information about patients’ detailed medical histories, present medication, and biochemical and echocardiography results. Follow-up information, including in-hospital mortality, readmission rate and short-term lipid profile alterations, was obtained via biobanks, telephones and questionnaires by the general practitioner (GP). Written informed consent was obtained from all study participants, with ethics committee approval at the First Affiliated Hospital of Xi’an Jiaotong University. The study was registered at ClinicalTrials.gov, ClinicalTrials.gov ID: NCT05184530.

### Study cohorts and treatments

Participants were allocated into four groups according to different lipid-lowering strategies: 1). Statin, 2). Statin plus ezetimibe, 3). Statin plus evolocumab and 4). Statin plus ezetimibe plus evolocumab, contingent on whether they began evolocumab during hospitalization. Patients were initiated with evolocumab (140 mg per 2 weeks, subcutaneous injection) during hospitalization either due to extremely high LDL levels and/or large area myocardial infarction based on the Chinese consensus on dyslipidemia management in patients with high-risk atherosclerosis disease [9]. The use of statin (20 mg atorvastatin or 10 mg rosuvastatin per day) or statin plus ezetimibe (10 mg per day) therapy was based on the present guidelines for the treatment of AMI [11].

### Propensity score match

Because patients received statin, ezetimibe and evolocumab according to their admission LDL levels and varied greatly in baseline characteristics, propensity score matching (PSM) was employed, and covariates were age, sex, admission blood pressure and lipid profiles. All eligible participants were (1) propensity-matched 1:2:2 of statin plus evolocumab vs. statin vs. statin plus ezetimibe and (2) propensity-matched 1:2:2 of statin plus ezetimibe evolocumab vs. statin vs. statin plus ezetimibe. The propensity score was determined by R 3.6.0 and the package MatchIt and calculated using the values of the covariates.

### Statistical analyses

All statistical analyses were performed by using SPSS for Mac 22.0 (SPSS Inc., Chicago, IL), GraphPad 9.0 Prism (GraphPad Software San Diego, CA) or R 3.6.0. Data are presented as frequencies or percentages for categorical variables and the mean ± SD for continuous variables, unless otherwise indicated. A simple t test was used to compare continuous variables that were normally distributed. The Mann–Whitney U test was used to compare continuous variables that did not conform to a normal distribution. The χ2 test was used to compare categorical variables. One-way ANOVA was used to compare continuous variables of three or more independent (unrelated) groups. A value of *P* < 0.05 was considered statistically significant.

## Results

### Study cohort

**Fig. 1:**
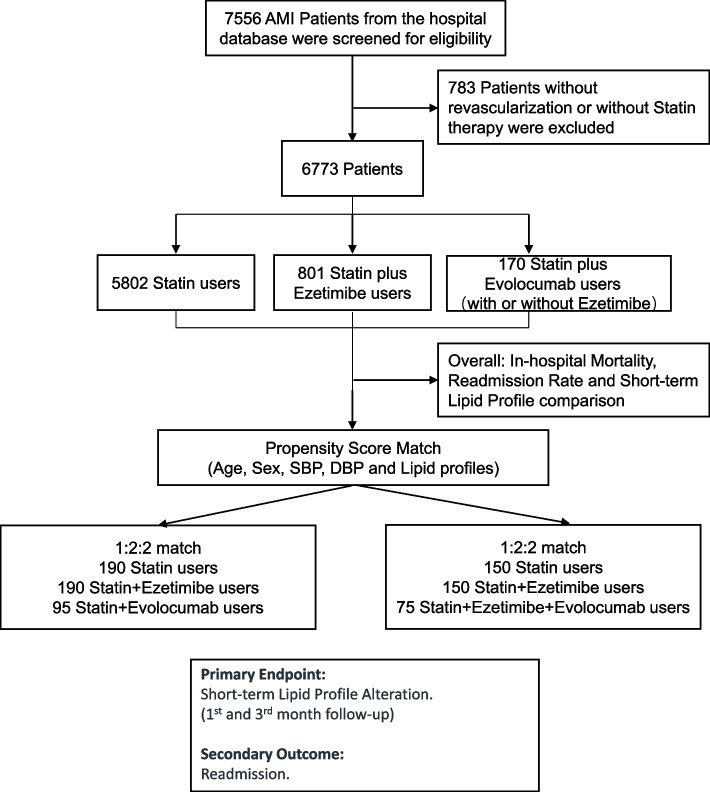
Patient selection, propensity score matching and follow-up

A total of 7556 AMI patients from the biobank database between January 2016 and December 2020 were screened for eligibility. After excluding those without revascularization or statin-based therapy, the remaining 5802 statin users, 801 statin plus ezetimibe users and 170 statin plus evolocumab users (including 95 users without and 75 users with ezetimibe) were selected for this study. Then, ^1−^ and 3-month follow-up data were collected and analyzed, including in-hospital mortality, readmission rate and lipid profiles (Fig. 1).

As admission LDL level and crowd size vary among different lipid-lowering strategy groups, propensity score matching (PSM) was performed for further analysis. ^First,^ PSM was based on statin plus evolocumab therapy, and of these, 95 users were successfully matched with 190 statin users and 190 statin plus ezetimibe users^.^ Second PSM was based on statin plus ezetimibe plus evolocumab (triple) therapy, and of these, 75 users were successfully matched with 150 statin users and 150 statin plus ezetimibe users. The matched groups were well balanced in terms of demographic and clinical characteristics (Appendix Tables 1 and 2, Appendix Figs. 1 and 2).

### Baseline characteristics and short-term follow-up in the whole cohort

**Fig. 2:**
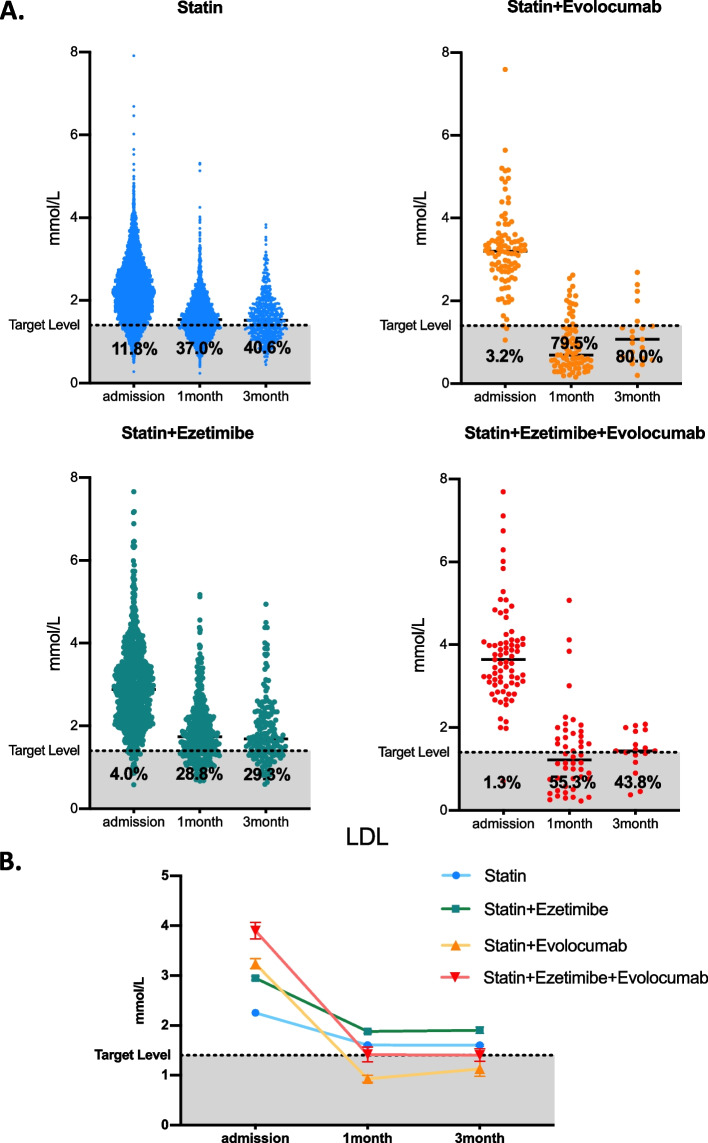
LDL level and compliance rate on admission and short-term follow-up among different lipid-lowering strategies in AMI patients. **A**. LDL level and compliance rate among different lipid-lowering strategies on admission and at the 1- and 3-month follow-ups. Each point represents one AMI patient’s lipid data. **B** LDL alterations among different lipid-lowering strategies on admission and at the 1st and 3rd month follow-ups. Data are shown as the mean ± SEM LDL Target Level = 1.4 mmol/L

In the whole cohort, the mean ages were 62.90 ± 11.91, 58.73 ± 12.16, 57.72 ± 11.07 and 54.38 ± 11.77 years among the statin, statin plus ezetimibe, statin plus evolocumab and triple therapy groups. The admission LDL levels were 2.25 ± 0.74, 2.95 ± 1.03, 3.24 ± 0.98 and 3.90 ± 1.45 mmol/L, respectively (Table 1), which is also inconsistent with the basic strategy that higher LDL levels require intensive lipid-lowering therapy in AMI patients. According to 2019 ESC/EAS Guidelines for the management of dyslipidemias [2], for patients at very high cardiovascular risk, LDL reduction of ≥50% from baseline and an LDL goal of < 1.4 mmol/L (< 55 mg/dL) are recommended. We further analyzed the control situation of LDL (< 1.4 mmol/L) among each group. On admission, the target rate was 11.8, 4.0, 3.2 and 1.3% among each group. In the 1-month follow-up, approximately 37.0, 28.8, 79.5 and 55.3% of all patients reached the treatment goal among each group, and after 3 months, 40.6, 29.3, 80.0 and 43.8% of all patients reached the treatment goal, respectively (Table 1). Despite higher LDL levels at admission, AMI patients achieved a promising control rate in short-term PCSK9i treatment Fig. 2.

**Table 1:** Distribution of demographic and clinical characteristics according to different lipid-lowering strategies in AMI patients

		Statin	Statin+ Ezetimibe	Statin+ Evolocumab	Statin+ Ezetimibe+ Evolocumab
**Age (years)**		62.90 ± 11.91	58.73 ± 12.16	57.72 ± 11.07	54.38 ± 11.77
**Female Sex(%)**		20.3	21.0	15.7	18.8
**SBP (mmHg)**		123.41 ± 22.24	125.20 ± 22.79	127.54 ± 22.20	122.72 ± 23.04
**DBP (mmHg)**		77.17 ± 14.53	79.01 ± 15.02	82.36 ± 14.49	78.98 ± 15.32
**EF(%)**		51.11 ± 10.27	52.81 ± 10.39	51.11 ± 10.27	51.27 ± 9.72
**Pro-BNP (pg/mL)**		2071.69	1334.93	2071.69	1135.26
**HbA1c(%)**		6.33 ± 1.45	6.29 ± 1.44	6.33 ± 1.45	6.75 ± 2.00
**Cre (umol/L)**		77.94	70.48	69.36	62.97
**UA (umol/L)**		333.91	340.59	330.43	330.08
**TG (mmol/L)**		1.48 ± 0.99	1.94 ± 0.66	1.57 ± 0.74	1.92 ± 1.02
**HDL (mmol/L)**		0.93 ± 0.22	0.98 ± 0.24	1.00 ± 0.21	1.00 ± 0.23
**TCDL (mmol/L)**		3.93 ± 0.99	4.76 ± 1.25	4.96 ± 1.19	5.69 ± 1.50
**ApoA(g/L)**		1.05 ± 0.19	1.09 ± 0.19	1.09 ± 0.17	1.09 ± 0.20
**ApoB(g/L)**		0.77 ± 0.20	0.95 ± 0.24	1.00 ± 0.23	1.16 ± 0.29
**ApoE (mg/L)**		35.64 ± 14.04	41.59 ± 18.67	41.96 ± 12.92	46.68 ± 19.88
**Lp(a) (mg/L)**		261.68	302.22	330.56	368.69
**Death (%)**		1.9	0.7	0.8	0.0
**Readmission (%)**		10.4	14.7	4.0	5.3
**LDL (mmol/L)**	**Admission**	2.25 ± 0.74	2.95 ± 1.03	3.24 ± 0.98	3.90 ± 1.45
	**Under Target**	11.8	4.0	3.2	1.3
	**Follow-up(1 m)**	1.60 ± 0.51	1.87 ± 0.75	0.93 ± 0.63	1.41 ± 1.02
	**Under Target**	37.0	28.8	79.5	55.3
	**Follow-up(3 m)**	1.60 ± 0.54	1.90 ± 0.82	1.13 ± 0.67	1.40 ± 0.50
	**Under Target**	40.6	29.3	80.0	43.8

Data are shown as the mean ± SD, median or n (%)

*SBP* Systolic blood pressure, *DBP* Diastolic blood pressure, *EF* Ejection fraction, *HbA1c* Hemoglobin A1c, *Cre* Creatinine, *UA* Uric acid, *TG* Triglyceride, *LDL* Low density lipoprotein, *HDL* High density lipoprotein, *ApoA* Apolipoprotein A, *ApoB* Apolipoprotein B, *ApoE* Apolipoprotein E, *TCDL* Total cholesterol lipoprotein, *Lp(a)* Lipoprotein(a)

Approximately 1.9% of AMI patients died with statin alone therapy, and 0.7, 0.8, and 0.0% died among the statin plus ezetimibe, evolocumab dual and triple groups, respectively, during the 3-month follow-up. The readmission rates were 4.0 and 5.3% with PCSK9i treatment and more than 10%, approximately 10.4% in statin users and 14.7% in statin plus ezetimibe users, respectively.

### Statin plus evolocumab therapy (dual therapy)-based PSM analysis

Ninety-five statin plus evolocumab users, 190 statin users and 190 statin plus ezetimibe users were well matched for this PSM analysis. The mean age was 59.42 ± 11.66, 58.62 ± 12.73 and 58.53 ± 10.63 among each group, and the admission LDL was 3.11 ± 1.02, 3.24 ± 1.13, and 3.24 ± 0.98 mmol/L, respectively, after PSM adjustments.

**Fig. 3:**
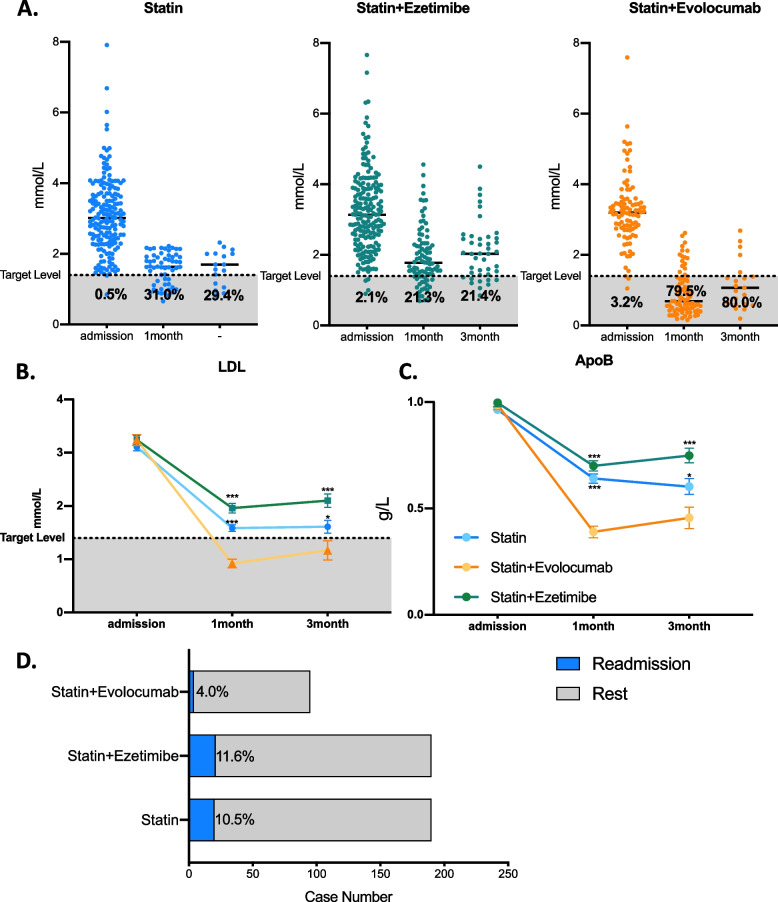
Lipid profile alterations and short-term follow-up among statin plus evolocumab/statin/statin plus ezetimibe lipid-lowering strategies in AMI patients after PSM adjustment. **A** LDL level and compliance rate among different lipid-lowering strategies on admission and ^at the 1-^ and 3-month follow-ups. Each point represents one AMI patient’s lipid data after PSM adjustment. LDL (**B**) and ApoB (**C**) alterations among different lipid-lowering strategies on admission and at the 1- and 3-month follow-ups. Data are shown as the mean ± SEM. **D** Readmission rate among each group. Ninety-five statin plus evolocumab users, 190 statin plus ezetimibe users and 190 statin users were chosen after 1:2:2 propensity score matching. For statistical analysis, one-way ANOVA followed by Sidak’s multiple comparison test was applied, * *P* < 0.05, ***P < 0.001

On admission, 0.5, 2.1 and 3.2% of AMI patients reached target LDL levels among the statin, statin plus ezetimibe, and statin plus evolocumab groups, respectively. At the 1-month follow-up, the target rates were 31.0, 21.3, and 79.5% and 29.4, 21.4, and 80.0% after 3 months, respectively (Fig. 3A). The mean LDL level was significantly decreased in statin plus evolocumab users compared to the other two groups, with 0.92 ± 0.62**,** 1.58 ± 0.44, and 1.96 ± 0.82 mmol/L in the 1st month and 1.17 ± 0.73, 1.61 ± 0.49, and 2.10 ± 0.82 months in the 3rd month. (Table 2 and Fig. 3.B). Additionally, a similar trend was observed in the ApoB level, with 0.39 ± 0.20, 0.64 ± 0.16, and 0.70 ± 0.22 g/L in the 1st month and 0.46 ± 0.20, 0.59 ± 0.15, and 0.75 ± 0.22 g/L in the 3rd month follow-up, respectively. Approximately 4.0% of AMI patients were rehospitalized with statin plus evolocumab therapy, and 11.6 and 10.5% readmission rates were observed in the other two groups during the short-term follow-up.

**Table 2:** Short-term lipid profile alteration among statin plus evolocumab/statin/statin plus ezetimibe lipid-lowering strategies in AMI patients after PSM adjustment

		Statin	Statin+ Ezetimibe	Statin+ Evolocumab	p1	p2
**LDL (mmol/L)**	Admission	3.11 ± 1.02	3.24 ± 1.13	3.24 ± 0.98	0.303	0.968
	Follow-up(1 m)	1.58 ± 0.44	1.96 ± 0.82	0.92 ± 0.62	< 0.001	< 0.001
	Follow-up(3 m)	1.61 ± 0.49	2.10 ± 0.82	1.17 ± 0.73	0.048	< 0.001
**TG (mmol/L)**	Admission	1.56 ± 1.01	1.55 ± 0.72	1.57 ± 0.74	0.927	0.823
	Follow-up(1 m)	1.33 ± 0.67	1.53 ± 0.70	1.29 ± 0.65	0.121	0.038
	Follow-up(3 m)	1.57 ± 1.13	1.66 ± 0.84	1.18 ± 0.51	0.214	0.011
**HDL (mmol/L)**	Admission	0.98 ± 0.22	1.02 ± 0.22	0.99 ± 0.21	0.593	0.364
	Follow-up(1 m)	1.01 ± 0.22	1.05 ± 0.25	1.03 ± 0.24	0.042	0.654
	Follow-up(3 m)	1.00 ± 0.18	1.00 ± 0.25	1.14 ± 0.32	0.145	0.078
**ApoA (g/L)**	Admission	1.08 ± 0.20	1.11 ± 0.20	1.09 ± 0.17	0.785	0.240
	Follow-up(1 m)	1.17 ± 0.18	1.18 ± 0.18	1.21 ± 0.19	0.223	0.390
	Follow-up(3 m)	1.20 ± 0.17	1.15 ± 0.20	1.25 ± 0.24	0.525	0.122
**ApoB** **(g/L)**	Admission	0.99 ± 0.21	1.00 ± 0.26	1.00 ± 0.23	0.676	0.974
	Follow-up(1 m)	0.64 ± 0.16	0.70 ± 0.22	0.39 ± 0.20	< 0.001	< 0.001
	Follow-up(3 m)	0.59 ± 0.15	0.75 ± 0.22	0.46 ± 0.20	0.038	< 0.001
**ApoE (mg/L)**	Admission	41.83 ± 18.16	41.60 ± 16.61	41.96 ± 12.92	0.950	0.853
	Follow-up(1 m)	33.82 ± 11.29	34.46 ± 11.93	20.79 ± 9.39	< 0.001	< 0.001
	Follow-up(3 m)	32.83 ± 10.80	30.44 ± 9.50	21.59 ± 8.18	0.018	0.013
**LP(a) (mg/L)**	Admission	331.77	310.66	330.55	0.975	0.679
	Follow-up(1 m)	281.96	283.66	271.18	0.902	0.876
	Follow-up(3 m)	358.29	370.88	345.92	0.951	0.867
**TCDL (mmol/L)**	Admission	4.97 ± 1.19	5.03 ± 1.30	5.00 ± 1.16	0.799	0.884
	Follow-up(1 m)	3.16 ± 0.58	3.57 ± 0.90	2.37 ± 0.74	< 0.001	< 0.001
	Follow-up(3 m)	3.18 ± 0.56	3.72 ± 1.02	2.79 ± 0.72	0.074	< 0.001

Data are shown as the mean ± SD, median or n (%). p1, Statin plus evolocumab vs. statin; p2, Statin plus evolocumab vs. statin plus ezetimibe. For statistical analysis, one-way ANOVA followed by Sidak’s multiple comparison test was applied

*TG* Triglyceride, *LDL* Low-density lipoprotein, *HDL* High-density lipoprotein, *ApoA* Apolipoprotein A, *ApoB* Apolipoprotein B, *ApoE* Apolipoprotein E, *TCDL* Total cholesterol lipoprotein, *Lp(a)* Lipoprotein(a)

### Statin plus ezetimibe plus evolocumab therapy (triple therapy)-based PSM analysis

In the 7556 AMI patients, 75 patients received triple therapy (statin plus ezetimibe plus evolocumab) and were well matched with 150 statin users and 150 statin plus ezetimibe users. The mean age was 52.59 ± 11.92, 53.57 ± 11.29 and 53.67 ± 11.86 among the groups, and the admission LDL levels were 3.59 ± 0.95, 3.82 ± 1.20, and 3.90 ± 1.45 mmol/L, respectively, after PSM adjustments.

**Fig. 4:**
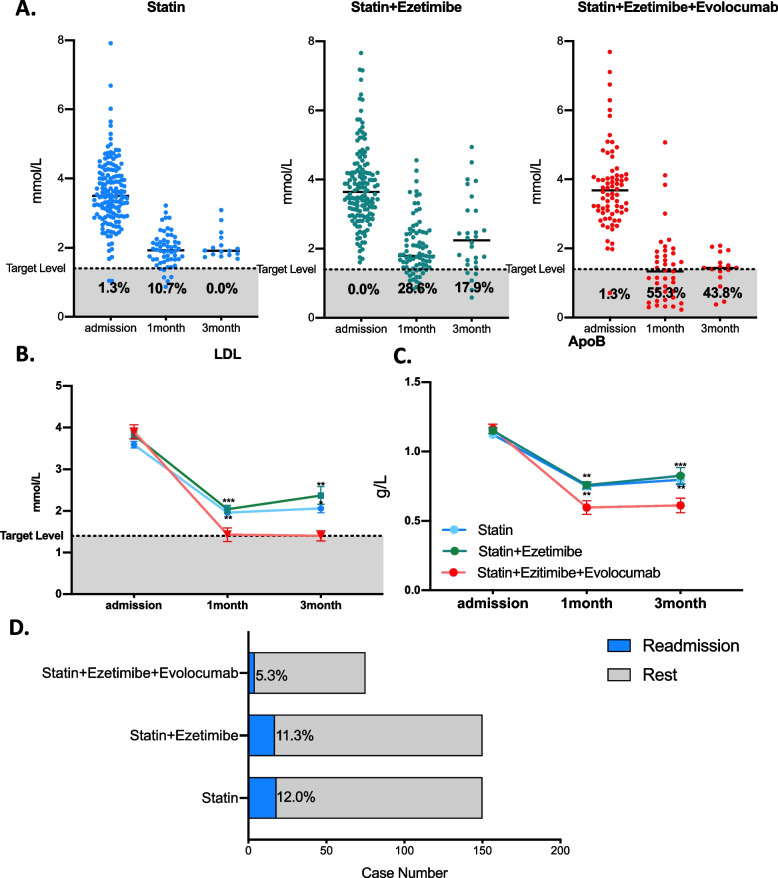
Lipid profile alterations and short-term follow-up among statin plus ezetimibe plus evolocumab/statin/statin plus ezetimibe lipid-lowering strategies in AMI patients after PSM adjustment. **A** LDL level and compliance rate among different lipid-lowering strategies on admission and at the 1- and 3-month follow-ups. Each point represents one AMI patient’s lipid data after PSM adjustment. LDL (**B**) and ApoB (**C**) alterations among different lipid-lowering strategies on admission and at the 1- and 3-month follow-ups. Data are shown as the mean ± SEM. **D** Readmission rate among each group. Seventy-five statin plus ezetimibe plus evolocumab users, 150 statin plus ezetimibe users and 150 statin users were chosen after 1:2:2 propensity score matching. For statistical analysis, one-way ANOVA followed by Sidak’s multiple comparison test was applied, * *P* < 0.05, ** *P* < 0.01, *** *P* < 0.001

Approximately 1.3, 0 and 1.3% of AMI patients reached target LDL levels among the statin, statin plus ezetimibe, and triple therapy groups during admission. At the 1-month follow-up, the achieved rates were 10.7, 28.6, and 55.3% and 0.0, 17.9, and 43.8% after 3 months, respectively (Fig. 4A). Additionally, the mean LDL level was significantly decreased in triple therapy patients compared to the other two groups, with 1.43 ± 1.06, 1.96 ± 0.49, and 2.04 ± 0.81 mmol/L in the 1st month and 1.40 ± 0.50, 2.06 ± 0.42, and 2.37 ± 1.13 mmol/L after the 3-month follow-up (Table 3 and Fig. 4.B). Additionally, a similar decrease was observed in ApoB levels, and triple medication users reached 0.59 ± 0.29 0.61 ± 0.20 g/L in the 1st month and 3rd month, compared to 0.75 ± 0.17 and 0.80 ± 0.13 g/L in the statin group and 0.76 ± 0.20 and 0.83 ± 0.29 g/L in the statin plus ezetimibe group. A total of 5.3% of AMI patients were rehospitalized with triple therapy, which was 6.7 and 6.0% lower than those in statin and statin plus ezetimibe users, respectively.

**Table 3:** Short-term lipid profile alteration among statin plus ezetimibe plus evolocumab/statin/statin plus ezetimibe lipid-lowering strategies in AMI patients after PSM adjustment

		Statin	Statin +Ezetimibe	Statin + Ezetimibe + Evolocumab	p1	p2
**LDL (mmol/L)**	Admission	3.59 ± 0.95	3.82 ± 1.20	3.90 ± 1.45	0.060	0.666
	Follow-up(1 m)	1.96 ± 0.49	2.04 ± 0.81	1.43 ± 1.06	0.001	0.001
	Follow-up(3 m)	2.06 ± 0.42	2.37 ± 1.13	1.40 ± 0.50	< 0.001	< 0.001
**TG (mmol/L)**	Admission	2.08 ± 1.12	1.92 ± 1.04	1.93 ± 1.01	0.311	0.948
	Follow-up(1 m)	1.72 ± 0.71	1.58 ± 0.68	1.50 ± 0.72	0.148	0.539
	Follow-up(3 m)	1.53 ± 0.64	1.82 ± 0.74	1.74 ± 1.17	0.534	0.796
**HDL (mmol/L)**	Admission	0.98 ± 0.24	1.01 ± 0.22	1.00 ± 0.23	0.472	0.818
	Follow-up(1 m)	0.99 ± 0.24	0.99 ± 0.21	0.94 ± 0.21	0.253	0.176
	Follow-up(3 m)	1.03 ± 0.29	1.00 ± 0.27	0.95 ± 0.18	0.316	0.491
**ApoA (g/L)**	Admission	1.09 ± 0.19	1.11 ± 0.20	1.09 ± 0.21	0.877	0.456
	Follow-up(1 m)	1.15 ± 0.20	1.14 ± 0.19	1.10 ± 0.18	0.850	0.336
	Follow-up(3 m)	1.11 ± 0.16	1.15 ± 0.23	1.12 ± 0.14	0.525	0.606
**ApoB (g/L)**	Admission	1.16 ± 0.29	1.15 ± 0.24	1.16 ± 0.29	0.269	0.730
	Follow-up(1 m)	0.75 ± 0.17	0.76 ± 0.20	0.59 ± 0.29	0.004	0.001
	Follow-up(3 m)	0.80 ± 0.13	0.83 ± 0.29	0.61 ± 0.20	0.008	0.010
**ApoE (mg/L)**	Admission	46.68 ± 19.88	46.90 ± 21.08	46.68 ± 19.88	0.825	0.939
	Follow-up(1 m)	31.00 ± 8.62	36.77 ± 16.37	28.92 ± 13.96	0.573	0.061
	Follow-up(3 m)	37.91 ± 10.10	34.56 ± 14.43	30.26 ± 14.06	0.265	0.466
**LP(a) (mg/L)**	Admission	331.77	375.71	433.87	0.982	0.248
	Follow-up(1 m)	450.00	283.66	271.18	0.587	0.851
	Follow-up(3 m)	435.08	427.27	595.43	0.502	0.332
**TCDL (mmol/L)**	Admission	5.55 ± 1.14	5.72 ± 1.30	5.79 ± 1.42	0.181	0.739
	Follow-up(1 m)	3.52 ± 0.65	3.55 ± 0.89	2.82 ± 1.18	< 0.001	< 0.001
	Follow-up(3 m)	3.65 ± 0.74	3.93 ± 1.39	2.97 ± 0.64	0.009	0.003

Data are shown as the mean ± SD, median or n (%). p1, Statin plus ezetimibe plus evolocumab vs. statin; p2, Statin plus ezetimibe plus evolocumab vs. statin plus ezetimibe. For statistical analysis, one-way ANOVA followed by Sidak’s multiple comparison test was applied

*TG* Triglyceride, *LDL* Low-density lipoprotein, *HDL* High-density lipoprotein, *ApoA* apolipoprotein A, *ApoB* Apolipoprotein B, *ApoE* Apolipoprotein E, *TCDL* Total cholesterol lipoprotein, Lp(a) Lipoprotein(a)

## Discussion

In this single-center, retrospective, population-based study, we found that in-hospital initiation of PCSK9 was effective in short-term lipid control among AMI patients. In both the PCSK9i plus statin therapy with or without ezetimibe cohort, the mean LDL levels decreased by more than 60%. In addition, more than 50% of patients in the above two groups reached the target LDL-C level after 1 month of treatment. PCSK9i plus statin therapy with or without ezetimibe exhibits a better lipid-lowering effect than statin or statin plus ezetimibe therapy.

Previously, PCSK9 inhibitors have been shown to reduce major cardiovascular events in secondary prevention and improve adherence and quality of life in high-risk patients with coronary artery disease [12, 13]. The EVOPACS study confirmed that compared with a high-intensity statin alone, the very early application of evolocumab brings a very significant reduction in LDL-C levels in ACS patients [14]. Additionally, the ODYSSEY study demonstrated that alirocumab added to intensive statin therapy reduced the occurrence of both Type 1 and Type 2 MI among patients after a recent ACS [15]. To the best of our knowledge, our study is the first to investigate lipid profile alterations as well as the short-term outcomes of PCSK9i initiated together with statins among AMI patients, focusing on real-world evidence. Similar to the EVOPACS study [14], our results show that LDL is significantly decreased via PCSK9i treatment after 1-month and 3-month follow-up when added to either statin or statin plus ezetimibe therapy. It is also noteworthy that ApoB, the primary apolipoprotein of LDL particles, exhibited the same trend as LDL among each treatment group, further proving the efficiency of PCSK9i in the lipid-lowering strategy.

In addition, our study shows a decreased readmission rate in the short-term follow-up via PCSK9i therapy. Mechanistically, previous studies have proven that after adjusting for ACSVD risk factors (including LDL level), serum PCSK9 levels were linearly associated with the fraction and amount of necrotic core tissue in coronary atherosclerosis [16]. Compared with healthy volunteers, a significantly higher concentration of PCSK9 was observed in patients with ACS [17]. Additionally, increased PCSK9 levels were associated with higher platelet reactivity and might be a possible predictor of ischemic events in ACS patients undergoing PCI [18]. Taken together, based on a 3-month follow-up, our results have provided further clinical benefits that PCSK9i might exert lasting cardiovascular benefits on AMI patients apart from lipid control. Long-term follow-up data are warranted to further validate the efficacy of the in-hospital initiation of PCSK9i [19].

Notably, in terms of lipid-lowering effects, we found that triple therapy with PCSK9i, statin and ezetimibe was not superior to statin plus evolocumab treatment before PSM adjustment. Several potential mechanisms may account for this phenomenon. First, most patients with triple treatment have extremely high LDL levels on admission, which was even 20% higher than that of the dual therapy group. Second, different lipid-lowering mechanisms also influence the effect. Ezetimibe inhibits the absorption of cholesterol from the small intestine and decreases the amount of cholesterol normally available to liver cells; the lower levels of cholesterol in the liver cells lead them to absorb more cholesterol from circulation and thus “indirectly” lower the levels of circulating cholesterol. On the other hand, evolocumab inhibits PCSK9 from binding to LDL receptors on the liver surface, and there are more LDL receptors on the surface of liver cells to remove LDL-C from the blood directly. Finally, limited therapy users were included in this study, and reduced compliance may also affect the results.

Via a 3-month follow-up, the strength of our study is to demonstrate that PCSK9i based therapy exhibits a better lipid-lowering effect, as well as a further clinical benefit on AMI patients apart from lipid control. However, there are several limitations in the current work. First, this study is limited in its single-center, retrospective and observational nature. Future multicenter studies based on longer follow-up are needed. Additionally, although the dosage and frequency of statins (20 mg atorvastatin or 10 mg rosuvastatin per day) are fixed, the data on whether statin therapy was started during or before hospital admission and whether statins were not allowed due to their side effects are inaccessible so that it is not possible to match this situation in the four groups. However, the initiation, dosage and frequency of evolocumab were fixed among all the groups in this study. Third, as alirocumab was not prescribed in this study, the results may not apply to patients on alirocumab treatment.

## Conclusions

In conclusion, through this retrospective, PSM-adjusted cohort study, we have further proved the efficiency and safety outcomes of PCSK9i during the short-term follow-up based on real-world analysis among AMI patients. The long-term effectiveness, as well as the specific mechanism of PCSK9i for reducing major cardiovascular events, remain to be explored.

## Supplementary Information

**Additional file 1: Fig. S1.** Jitter and hist plots (statin plus evolocumab-based PSM).

**Additional file 2: Fig. S2.** Jitter and hist plots (triple therapy-based PSM).

**Additional file 3: Table. S1.** Summary of statin plus evolocumab therapy-based PSM.

**Additional file 4: Table. S2.** Summary of triple therapy-based PSM.

## Data Availability

The datasets used or analyzed during the current study are available from the corresponding author upon reasonable request.
