# Supplementary material for: In-hospital initiation of PCSK9 inhibitor and short-term lipid control in patients with acute myocardial infarction
Source: Lipids Health Dis. 2022 Oct 24;21:105. doi: 10.1186/s12944-022-01724-9 (PMC9590135; doi:10.1186/s12944-022-01724-9)

Statin+Evolocumab Vs. Statin

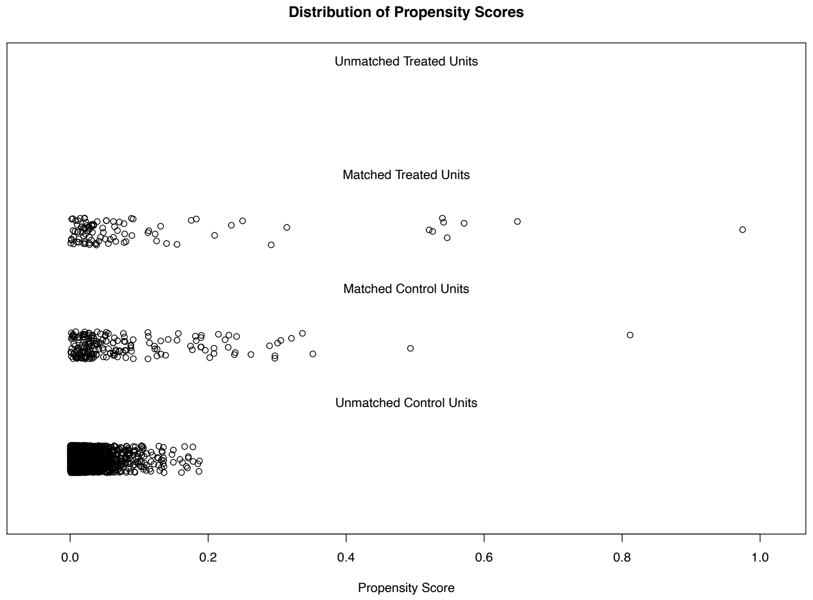

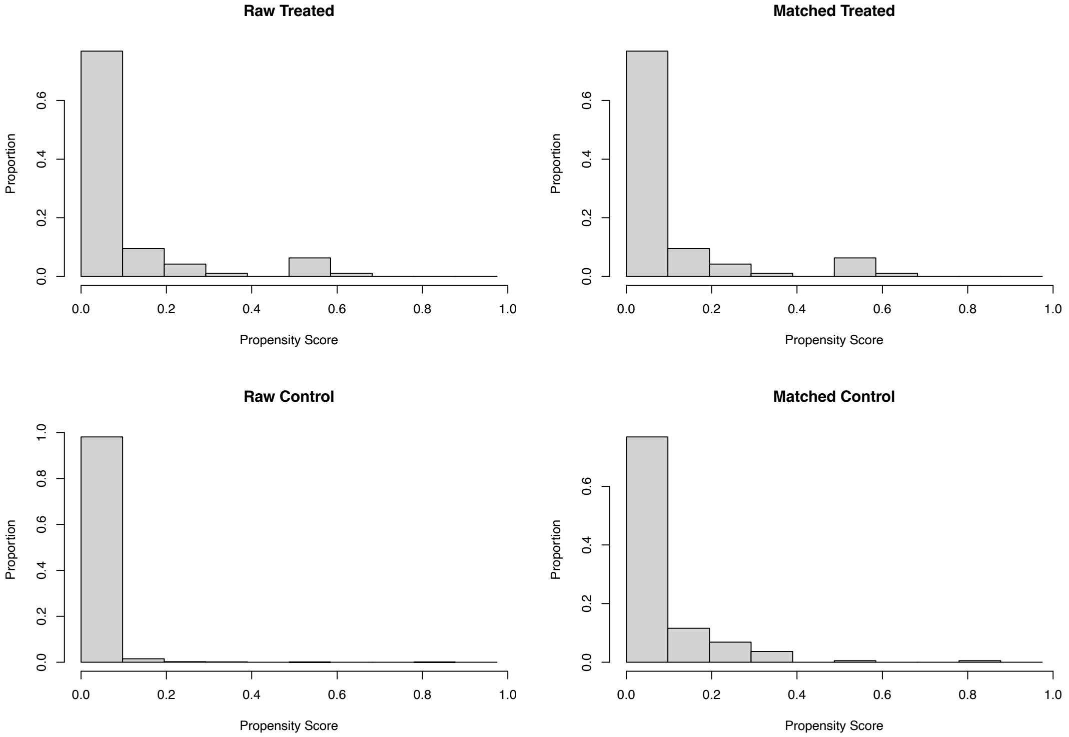

Statin+Evolocumab Vs. Statin+Ezetimibe

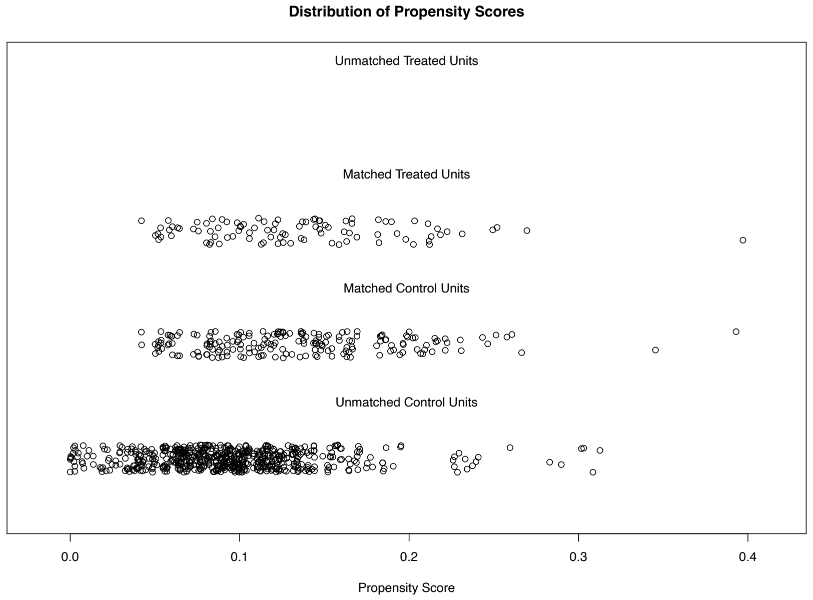

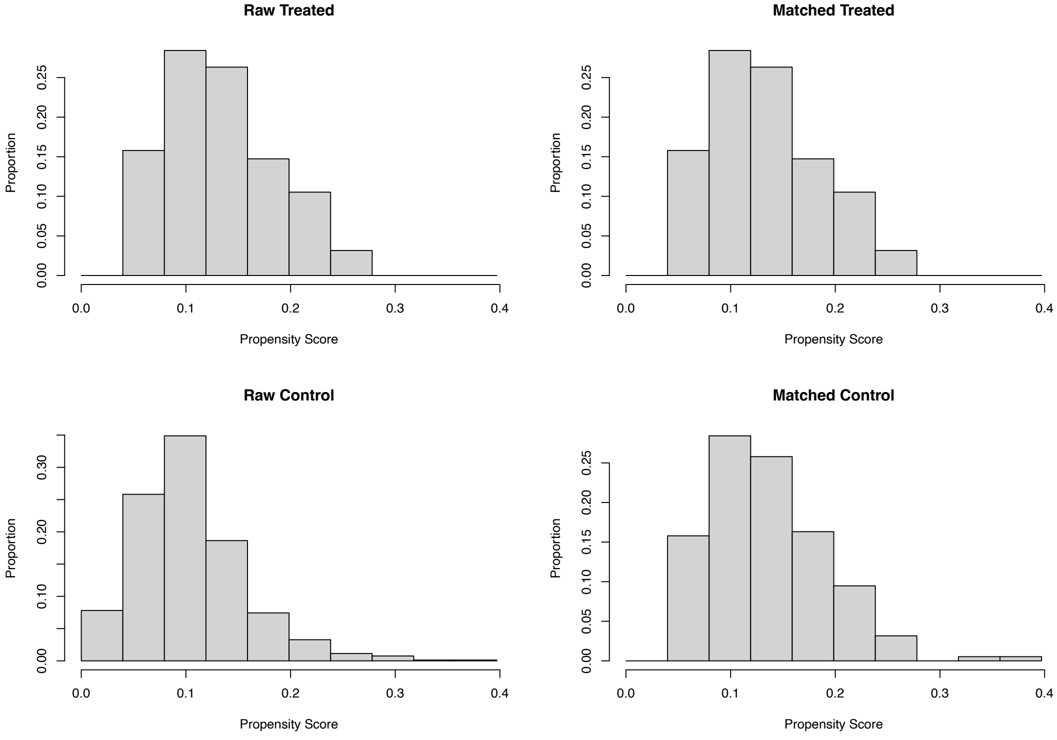

Supplement: Supplementary file 1 — Additional file 1: Fig. S1. Jitter and hist plots (statin plus evolocumab-based PSM). [file 12944_2022_1724_MOESM1_ESM.pdf]
