# Supplementary material for: In-hospital initiation of PCSK9 inhibitor and short-term lipid control in patients with acute myocardial infarction
Source: Lipids Health Dis. 2022 Oct 24;21:105. doi: 10.1186/s12944-022-01724-9 (PMC9590135; doi:10.1186/s12944-022-01724-9)
Supplement: Supplementary file 3 — Additional file 3: Table. S1. Summary of statin plus evolocumab therapy-based PSM. [file 12944_2022_1724_MOESM3_ESM.docx]

**Statin+Evolocumab Vs. Statin**

Summary of Balance for All Data:

Means Treated Means Control Std. Mean Diff. Var. Ratio eCDF Mean eCDF Max

distance 0.1038 0.0157 0.5148 34.4472 0.3278 0.5597

Age 58.5263 62.5868 -0.3779 0.8170 0.0537 0.1817

Sex 0.1684 0.1978 -0.0786 . 0.0294 0.0294

SBP 126.8000 123.5633 0.1377 1.1376 0.0244 0.0872

DBP 81.1474 77.3836 0.2441 1.1381 0.0413 0.1313

LDL 3.2368 2.2845 0.9715 1.8415 0.2229 0.5284

TG 1.5715 1.4939 0.1046 0.6063 0.0338 0.1523

HDL 0.9971 0.9325 0.3151 0.8409 0.0436 0.2028

APOA 1.0862 1.0530 0.1977 0.7476 0.0269 0.1110

APOB 1.0031 0.7651 1.0403 1.3193 0.1795 0.4695

APOE 41.9611 35.6965 0.4850 0.8794 0.1006 0.3433

LPA 330.5579 261.6522 0.2229 1.7077 0.0567 0.1414

TCDL 5.0068 3.9273 0.9277 1.4377 0.1948 0.4758

Summary of Balance for Matched Data:

Means Treated Means Control Std. Mean Diff. Var. Ratio eCDF Mean eCDF Max

distance 0.1038 0.0771 0.1562 2.8823 0.0005 0.0789

Age 58.5263 59.4158 -0.0828 0.8656 0.0210 0.0842

Sex 0.1684 0.1632 0.0141 . 0.0053 0.0053

SBP 126.8000 128.1053 -0.0555 1.0307 0.0149 0.0737

DBP 81.1474 82.6000 -0.0942 1.1141 0.0202 0.0895

LDL 3.2368 3.1058 0.1337 0.9156 0.0458 0.1368

TG 1.5715 1.5607 0.0145 0.5370 0.0234 0.0895

HDL 0.9971 0.9824 0.0716 0.8329 0.0211 0.1211

APOA 1.0862 1.0798 0.0382 0.7321 0.0228 0.0789

APOB 1.0031 0.9916 0.0502 1.1525 0.0240 0.0842

APOE 41.9611 41.8289 0.0102 0.5061 0.0278 0.1789

LPA 330.5579 331.7684 -0.0039 1.0615 0.0176 0.0474

TCDL 5.0068 4.9691 0.0325 0.9606 0.0181 0.0684

Std. Pair Dist.

distance 0.1570

Age 1.1515

Sex 0.7735

SBP 1.0809

DBP 1.0596

LDL 0.5514

TG 1.1747

HDL 1.0970

APOA 1.1999

APOB 0.5753

APOE 1.1317

LPA 0.9007

TCDL 0.6732

Percent Balance Improvement:

Std. Mean Diff. Var. Ratio eCDF Mean eCDF Max

distance 69.7 70.1 99.8 85.9

Age 78.1 28.6 61.0 53.7

Sex 82.1 . 82.1 82.1

SBP 59.7 76.6 38.9 15.5

DBP 61.4 16.5 51.1 31.8

LDL 86.2 85.6 79.5 74.1

TG 86.1 -24.3 30.8 41.3

HDL 77.3 -5.5 51.6 40.3

APOA 80.7 -7.2 15.1 28.9

APOB 95.2 48.8 86.6 82.1

APOE 97.9 -429.8 72.3 47.9

LPA 98.2 88.8 69.0 66.5

TCDL 96.5 88.9 90.7 85.6

Sample Sizes:

Control Treated

All 5409 95

Matched 190 95

Unmatched 5219 0

Discarded 0 0

**Statin+Evolocumab Vs. Statin+Ezetimibe**

Summary of Balance for All Data:

Means Treated Means Control Std. Mean Diff. Var. Ratio eCDF Mean eCDF Max

distance 0.1342 0.1036 0.5117 1.2587 0.1555 0.2544

Age 58.5263 58.6222 -0.0089 0.7856 0.0241 0.0678

Sex 0.1684 0.2128 -0.1187 . 0.0444 0.0444

SBP 126.8000 124.6776 0.0903 1.0686 0.0226 0.0861

DBP 81.1474 78.8526 0.1488 1.0508 0.0298 0.0963

LDL 3.2368 2.9505 0.2921 0.8996 0.0750 0.2215

TG 1.5715 1.9452 -0.5035 0.1982 0.0500 0.0968

HDL 0.9971 0.9802 0.0822 0.7434 0.0257 0.1195

APOA 1.0862 1.0896 -0.0200 0.7656 0.0171 0.0482

APOB 1.0031 0.9451 0.2534 0.8990 0.0473 0.1630

APOE 41.9611 41.6636 0.0230 0.4773 0.0467 0.1951

LPA 330.5579 299.3338 0.1010 1.1992 0.0404 0.1054

TCDL 5.0068 4.7776 0.1970 0.8806 0.0553 0.1579

Summary of Balance for Matched Data:

Means Treated Means Control Std. Mean Diff. Var. Ratio eCDF Mean eCDF Max

distance 0.1342 0.1335 0.0113 1.0685 0.0019 0.0368

Age 58.5263 59.5421 -0.0945 0.7301 0.0326 0.0895

Sex 0.1684 0.1684 0.0000 . 0.0000 0.0000

SBP 126.8000 126.9158 -0.0049 0.9911 0.0176 0.0789

DBP 81.1474 81.2211 -0.0048 1.0384 0.0146 0.0737

LDL 3.2368 3.2424 -0.0056 0.7516 0.0504 0.1263

TG 1.5715 1.5509 0.0277 1.0513 0.0210 0.0789

HDL 0.9971 1.0219 -0.1214 0.8398 0.0276 0.1421

APOA 1.0862 1.1142 -0.1664 0.7171 0.0307 0.0895

APOB 1.0031 1.0020 0.0046 0.7551 0.0326 0.1000

APOE 41.9611 41.6005 0.0279 0.6044 0.0364 0.1895

LPA 330.5579 315.6579 0.0482 1.2719 0.0197 0.0632

TCDL 5.0068 5.0300 -0.0199 0.7989 0.0362 0.0947

Std. Pair Dist.

distance 0.0171

Age 1.3661

Sex 0.2737

SBP 1.0952

DBP 1.0630

LDL 0.9521

TG 1.0536

HDL 1.1473

APOA 1.2153

APOB 0.9849

APOE 1.0819

LPA 0.9773

TCDL 1.0138

Percent Balance Improvement:

Std. Mean Diff. Var. Ratio eCDF Mean eCDF Max

distance 97.8 71.2 98.8 85.5

Age -959.8 -30.4 -34.9 -31.9

Sex 100.0 . 100.0 100.0

SBP 94.5 86.5 21.9 8.3

DBP 96.8 23.9 50.9 23.5

LDL 98.1 -169.9 32.8 43.0

TG 94.5 96.9 57.9 18.4

HDL -47.7 41.1 -7.4 -18.9

APOA -731.5 -24.5 -79.6 -85.7

APOB 98.2 -163.8 31.1 38.6

APOE -21.2 31.9 21.9 2.9

LPA 52.3 -32.4 51.3 40.1

TCDL 89.9 -76.6 34.6 40.0

Sample Sizes:

Control Treated

All 794 95

Matched 190 95

Unmatched 604 0

Discarded 0 0
