# Supplementary material for: In-hospital initiation of PCSK9 inhibitor and short-term lipid control in patients with acute myocardial infarction
Source: Lipids Health Dis. 2022 Oct 24;21:105. doi: 10.1186/s12944-022-01724-9 (PMC9590135; doi:10.1186/s12944-022-01724-9)
Supplement: Supplementary file 4 — Additional file 4: Table. S2. Summary of triple therapy-based PSM. [file 12944_2022_1724_MOESM4_ESM.docx]

**Statin+Ezetimibe+Evolocumab Vs. Statin**

Summary of Balance for All Data:

Means Treated Means Control Std. Mean Diff. Var. Ratio eCDF Mean eCDF Max

distance 0.1770 0.0107 0.6338 63.9968 0.4203 0.7121

Age 53.6667 62.6193 -0.7551 0.9956 0.1180 0.3337

Sex 0.1867 0.1980 -0.0291 . 0.0113 0.0113

SBP 123.5467 123.3651 0.0079 1.0855 0.0143 0.0643

DBP 79.6400 77.2520 0.1531 1.1721 0.0252 0.1159

LDL 3.8985 2.2487 1.1371 3.8170 0.3207 0.6579

TG 1.9257 1.4847 0.4345 1.0567 0.0965 0.2477

HDL 1.0020 0.9319 0.3025 1.0727 0.0470 0.1342

APOA 1.0856 1.0524 0.1596 1.1467 0.0256 0.0783

APOB 1.1635 0.7696 1.3724 2.0274 0.2630 0.6526

APOE 46.6760 35.6547 0.5545 1.9992 0.1459 0.3505

LPA 433.8667 261.4010 0.4678 2.4116 0.1414 0.3263

TCDL 5.7853 3.9438 1.2997 2.0826 0.3020 0.6091

Summary of Balance for Matched Data:

Means Treated Means Control Std. Mean Diff. Var. Ratio eCDF Mean eCDF Max

distance 0.1770 0.1175 0.2266 3.3546 0.0009 0.1200

Age 53.6667 52.5867 0.0911 0.9920 0.0205 0.1267

Sex 0.1867 0.1667 0.0513 . 0.0200 0.0200

SBP 123.5467 122.2333 0.0575 1.3443 0.0201 0.0933

DBP 79.6400 78.9600 0.0436 1.1547 0.0154 0.0667

LDL 3.8985 3.5940 0.2099 2.3406 0.0275 0.1267

TG 1.9257 2.0821 -0.1541 0.8151 0.0294 0.1867

HDL 1.0020 0.9780 0.1036 0.9491 0.0212 0.0867

APOA 1.0856 1.0899 -0.0205 1.2147 0.0235 0.0733

APOB 1.1635 1.1255 0.1324 1.7548 0.0195 0.0933

APOE 46.6760 46.1280 0.0276 1.4977 0.0272 0.1133

LPA 433.8667 435.0800 -0.0033 0.9287 0.0544 0.1400

TCDL 5.7853 5.5506 0.1657 1.5481 0.0276 0.1333

Std. Pair Dist.

distance 0.2282

Age 1.1156

Sex 0.7357

SBP 1.0263

DBP 1.0992

LDL 0.4913

TG 1.0713

HDL 1.0998

APOA 1.0531

APOB 0.5328

APOE 0.8378

LPA 1.0636

TCDL 0.6058

Percent Balance Improvement:

Std. Mean Diff. Var. Ratio eCDF Mean eCDF Max

distance 64.2 70.9 99.8 83.1

Age 87.9 -83.6 82.6 62.0

Sex -76.7 . -76.7 -76.7

SBP -623.3 -260.8 -41.0 -45.3

DBP 71.5 9.4 38.9 42.5

LDL 81.5 36.5 91.4 80.7

TG 64.5 -270.8 69.5 24.6

HDL 65.8 25.5 54.9 35.4

APOA 87.1 -42.1 8.4 6.3

APOB 90.4 20.4 92.6 85.7

APOE 95.0 41.7 81.4 67.7

LPA 99.3 91.6 61.5 57.1

TCDL 87.3 40.4 90.9 78.1

Sample Sizes:

Control Treated

All 5763 75

Matched 150 75

Unmatched 5613 0

Discarded 0 0

**Statin+Ezetimibe+Evolocumab Vs. Statin+Ezetimibe**

Summary of Balance for All Data:

Means Treated Means Control Std. Mean Diff. Var. Ratio eCDF Mean eCDF Max

distance 0.1846 0.0770 0.7224 3.2200 0.2868 0.4258

Age 53.6667 58.6222 -0.4180 0.9565 0.0800 0.2045

Sex 0.1867 0.2128 -0.0672 . 0.0262 0.0262

SBP 123.5467 124.6776 -0.0495 1.0092 0.0170 0.0791

DBP 79.6400 78.8526 0.0505 1.0756 0.0173 0.0595

LDL 3.8985 2.9505 0.6534 1.9707 0.1894 0.3516

TG 1.9257 1.9452 -0.0192 0.3706 0.0358 0.1226

HDL 1.0020 0.9802 0.0941 0.9495 0.0254 0.0726

APOA 1.0856 1.0896 -0.0191 1.1724 0.0248 0.0688

APOB 1.1635 0.9451 0.7608 1.4156 0.1540 0.3918

APOE 46.6760 41.6636 0.2522 1.1302 0.0835 0.1958

LPA 433.8667 299.3338 0.3649 1.7066 0.1389 0.2911

TCDL 5.7853 4.7776 0.7112 1.3053 0.1846 0.3409

Summary of Balance for Matched Data:

Means Treated Means Control Std. Mean Diff. Var. Ratio eCDF Mean eCDF Max

distance 0.1846 0.1728 0.0793 1.4164 0.0031 0.0800

Age 53.6667 53.5733 0.0079 1.1032 0.0161 0.0533

Sex 0.1867 0.1800 0.0171 . 0.0067 0.0067

SBP 123.5467 124.7800 -0.0540 1.1501 0.0238 0.1000

DBP 79.6400 79.9600 -0.0205 1.0098 0.0141 0.0933

LDL 3.8985 3.8198 0.0543 1.4575 0.0160 0.0667

TG 1.9257 1.9163 0.0093 0.9522 0.0241 0.1067

HDL 1.0020 1.0093 -0.0317 1.1013 0.0329 0.1400

APOA 1.0856 1.1073 -0.1045 1.0293 0.0318 0.1067

APOB 1.1635 1.1508 0.0441 1.3798 0.0186 0.0733

APOE 46.6760 46.8993 -0.0112 0.8887 0.0209 0.0800

LPA 433.8667 375.7133 0.1577 1.1186 0.0806 0.2000

TCDL 5.7853 5.7221 0.0446 1.1920 0.0186 0.0600

Std. Pair Dist.

distance 0.0872

Age 0.9368

Sex 0.9068

SBP 1.1033

DBP 1.0916

LDL 0.7129

TG 1.0691

HDL 1.0877

APOA 1.0935

APOB 0.6350

APOE 0.9990

LPA 0.9166

TCDL 0.7635

Percent Balance Improvement:

Std. Mean Diff. Var. Ratio eCDF Mean eCDF Max

distance 89.0 70.2 98.9 81.2

Age 98.1 -120.6 79.8 73.9

Sex 74.5 . 74.5 74.5

SBP -9.1 -1419.2 -39.9 -26.4

DBP 59.4 86.6 18.1 -56.9

LDL 91.7 44.5 91.5 81.0

TG 51.4 95.1 32.6 13.0

HDL 66.4 -86.2 -29.4 -92.8

APOA -447.2 81.8 -28.0 -55.0

APOB 94.2 7.4 87.9 81.3

APOE 95.5 3.7 74.9 59.1

LPA 56.8 79.0 41.9 31.3

TCDL 93.7 34.1 89.9 82.4

Sample Sizes:

Control Treated

All 794 75

Matched 150 75

Unmatched 644 0

Discarded 0 0
